# Talking about Complimentary and Alternative Medicine: A Conversation Analysis Study

**DOI:** 10.1093/geroni/igab046.3053

**Published:** 2021-12-17

**Authors:** Sean Halpin, Kathleen Len, Michael Konomos

**Affiliations:** 1 University of Georgia, Decatur, Georgia, United States; 2 University of California Los Angeles, University of California, Los Angeles, California, United States; 3 Emory University, Atlanta, Georgia, United States

## Abstract

Multiple Myeloma (MM) is a clonal plasma cell malignancy characterized by low blood counts and increased risk of infection, and primarily afflicts older adults. Although MM is incurable, advances in treatment, including autologous stem cell transplant (ASCT) has improved the lifespan of patients. MM patients commonly use over-the-counter complementary and alternative medicines (CAM) alongside conventional cancer therapies which, often without recognition by health care practitioners, may impact their treatment. Using data from an 18-month ethnographic study, we applied conversation analysis to examine 1180 minutes of audio-recordings to describe how patients and nurses interacted about CAM during ASCT education visits. Patients (n=12) had a median age of 62 years (IQR= 54-73), were mostly white (n=12, 75%), male (n=9, 56%), and had a moderate score on the FACT-G7 of 15 (IQR= 10-20). All patients had a caregiver present during their visit. Nurses (n=3) were aged 39 (IQR= 29-49) all with at least five years providing care to patients with blood cancers. Results suggested that nurses rarely provided direct feedback about CAM modalities, instead providing brief responses, and moving on to other topics. Excerpts were categorized into three groups, (1) demonstration of implicit epistemic authority, (2) demonstration of deferred epistemic authority in patient-initiated conversations, and (3) demonstration of deferred epistemic authority in nurse-initiated conversations. Understanding how conversations surrounding CAM are navigated can provide insights into patient-communication in general, and methods for improving ASCT education.

